# Long noncoding RNA TCONS‐00106987 promotes atrial electrical remodelling during atrial fibrillation by sponging miR‐26 to regulate *KCNJ2*

**DOI:** 10.1111/jcmm.15869

**Published:** 2020-09-20

**Authors:** Juanjuan Du, Zhan Li, Xiao Wang, Jianhua Li, Donglu Liu, Ximin Wang, Jinqiu Wei, Shenzhou Ma, Yujiao Zhang, Yinglong Hou

**Affiliations:** ^1^ Department of Cardiology Shandong Provincial Qianfoshan Hospital Cheeloo College of Medicine Shandong University Jinan China; ^2^ Department of Critical Care Medicine The First Affiliated Hospital of Xiamen University Xiamen China

**Keywords:** atrial fibrillation, electrical remodelling, KCNJ2, long noncoding RNA, TCONS‐00106987

## Abstract

Long noncoding RNAs (lncRNAs) have been suggested to play indispensable roles in multiple heart diseases. However, the correlations between lncRNAs and atrial fibrillation (AF) are unclear. In this study, we performed comprehensive lncRNA profiling via high‐throughput RNA sequencing analysis using non‐AF and AF rabbit models. Based on a series of filtering pipelines and bioinformatics analyses, TCONS‐00106987 was selected for further research. TCONS‐00106987 levels were increased in the atria during AF. Moreover, the atrial effective refractory period was shortened and the AF inducibility was increased in vivo in response to lentiviral‐mediated up‐regulation of TCONS‐00106987. TCONS‐00106987 repression resulted in the opposite effects. Further studies indicated that TCONS‐00106987 expression was positively correlated with the expression of the protein‐coding gene *KCNJ2*. Luciferase reporter assays and whole‐cell patch‐clamp recording confirmed that TCONS‐00106987 promoted electrical remodelling via endogenous competition with microRNA‐26 (miR‐26) to induce transcription of its target gene *KCNJ2,* thereby increasing inward‐rectifier K^+^ current (I_K1_). In conclusion, our study reveals a pathogenic lncRNA‐miRNA regulatory network specific to atrial electrical remodelling that offers potential therapeutic targets for AF.

## 1 INTRODUCTION

Atrial fibrillation (AF), the most common sustained arrhythmia observed in clinical practice, results in high morbidity and mortality.^1^ Current therapies for AF present limitations such as high recurrence, poor tolerance and possible adverse effects.^2^ AF is an end result of the atrial remodelling that can occur due to various cardiac disorders.^3^ However, AF itself can also induce atrial remodelling, which contributes to the progression of arrhythmia.^4^ There are three principal pathophysiological mechanisms that contribute to AF, namely electrical remodelling, structural remodelling and autonomic neural remodelling.^4^, ^5^ Electrical remodelling is a significant component of atrial remodelling that occurs early in AF and results in a shortening of both the atrial effective refractory period (AERP) and the action potential duration (APD).^6^, ^7^ A large number of studies have shown that left atrial remodelling plays an important role in the occurrence and development of AF, but the roles and mechanisms of right atrial remodelling in AF have not been fully elucidated.

Long noncoding RNAs (lncRNAs) are a heterogeneous class of transcripts that range from 200 nt to 100 kb in length and do not possess protein‐coding potentials.^8^ Numerous studies have shown that lncRNAs may be involved in multiple cellular processes, including proliferation, differentiation, cell cycle regulation and apoptosis. From a functional perspective, lncRNAs are involved in gene expression and cellular activity through a variety of mechanisms such as chromatin remodelling, titration of transcription factors, sponging of microRNAs (miRNAs), alternative splicing and translational regulation of mRNAs.^9^, ^10^, ^11^, ^12^ Dysregulation of lncRNAs has been found to be associated with a number of heart diseases, including myocardial infarction, cardiac hypotrophy and heart failure^13^, ^14^, ^15^, ^16^ This suggests a potential use for these lncRNAs as therapeutic targets and/or biomarkers. However, the potential roles and mechanisms of lncRNAs in the context of AF remain poorly studied.

To provide a basis for the further study of the molecular pathogenesis of right atrial electrical remodelling in AF and to identify potential therapeutic targets for this disease, we compared the expression profiles of lncRNAs in AF and non‐AF rabbit right atria (RA) through the use of high‐throughput RNA sequencing. The function of lncRNA TCONS‐00106987, which is related to electrical remodelling, was investigated in vitro and in vivo using silencing and overexpression strategies. Additionally, we explored the mechanisms underlying the actions of TCONS‐00106987 in regard to the electrical remodelling that occurs during AF.

## 2 MATERIALS AND METHODS

### 2.1 Experimental animals

All animal experiments were performed in accordance with the guidelines approved by the Institutional Animal Care and Use Committee of Shandong Provincial Qianfoshan Hospital. All animal care adhered to the Guidelines for the Care and Use of Laboratory Animals (NIH Publication, 8th Edition, 2011).

### 2.2 Rabbit AF models

Twelve adult New Zealand white rabbits of either sex (weighing 2‐3 kg) were randomized into two groups that included sham‐operated (control) and AF groups (n = 6 per subgroup). The detailed procedures for establishing a rabbit model of AF are described in a previous study.^17^ Briefly, after anaesthetizing the rabbits with sodium pentobarbital (30 mg/kg), electrode leads were inserted into the right atrial appendage via the right jugular vein to achieve permanent pacing, and a pacemaker (AOO, made in Shanghai Fudan University, China) attached to the electrode lead was then implanted in a subcutaneous pocket. The AF group was subjected to continuous pacing (600 beats/min) for 7 days. The control group was treated using the same experimental procedures without pacing.

### 2.3 In vivo electrophysiological study

Atrial effective refractory period and AF inducibility were examined before and 7 days after pacing, as previously described.^17^, ^18^, ^19^ S1‐S2‐programmed electrical stimulation (PES) was used to detect the AERP by installing a catheter into the RA. The S1‐S2 intervals were started at 120 ms and followed by 5 ms decrements (S1:S2 = 8:1). The AERP was defined as the longest interval failing to propagate a response. PES with burst stimulation (120 ms) was used to induce AF, and a rapid irregular atrial rhythm lasting longer than 30 s was defined as a successful induction.

### 2.4 High‐throughput RNA‐seq

An Illumina Hiseq 2500 (Illumina, San Diego, CA, USA) was used to perform high‐throughput RNA‐Seq. The following standards were used to screen candidate lncRNAs: RNA length ≥ 200 nt, CPC score ≤ 0, CPAT probability ≤ 0.364 and phyloCSF score ≤‐20. Fragments per kilobase of transcript per million fragments mapped (FPKM) values were used to calculate the expression levels of the transcripts. Differentially expressed transcripts (DETs) were identified based on a *P* < .05 and a fold change > 2 times according to their FPKM values.^18^, ^19^

### 2.5 Bioinformatics analysis

For bioinformatics analysis, Gene Ontology (GO) and Kyoto Encyclopedia of Genes and Genomes (KEGG) pathway analyses were used to define the functions of differentially expressed genes in the GO vocabularies and graphical diagrams of biochemical pathways. Cis‐ and trans‐predictions were performed to identify the target genes of differentially expressed lncRNAs.^18^, ^20^ According to the lncRNA sequences, the shared binding sites between lncRNAs and miRNAs were then predicted according to the miRDB4.0, RNAhybrid, PITA and Ensemble BLAST. The TargetScan and RegRNA 2.0 databases were used to predict the target genes of relevant miRNAs. Subsequently, the connections between the target lncRNAs and miRNAs were confirmed by loss‐of‐function and gain‐of‐function experiments.

### 2.6 Isolation and culture of primary cardiomyocytes

The atria were removed from rabbits within 24 hours of birth and cut into 1 mm^3^ pieces. The pieces were then digested in petri dishes containing 1 mL collagenase II (0.1%) (Worthington, New Jersey, USA) and 4 mL of Dulbecco's modified Eagle's medium/Ham's F‐12 50/50 Mix (DMEM/F12) (Hyclone, Logan, Utah) in an incubator (37°C and 5% CO_2_). Then, the tissue was disrupted by titration using a pipette after 24 hours of digestion. The supernatant was centrifuged at 111 *g* for 5 min. The cells were resuspended in DMEM/F12 supplemented with 10% foetal bovine serum (Gibco, Auckland, New Zealand), 100 μg/mL penicillin and 100 μg/mL streptomycin (Hyclone, Logan, UT, USA) in a humidified incubator (37°C and 5% CO_2_). Cardiomyocytes were purified by means of differential attachment technique.^21^

### 2.7 RNA isolation and reverse transcription quantitative polymerase chain reaction (qRT‐PCR) analysis

Total RNA was extracted from cells and tissues using TRIzol reagent (Invitrogen, Carlsbad, USA). RNA concentrations were determined using a spectrophotometer (Merinton, SMA4000, USA). First‐strand cDNA was prepared using a PrimeScript^™^ RT Reagent Kit with gDNA Eraser (TAKARA, Dalian, China). The relative expression levels of target genes were assessed using quantitative real‐time PCR with SYBR Green (TAKARA, Dalian, China) on an ABI ViiA 7 Real‐Time PCR system (Applied Biosystems, Foster City, CA). miRNA quantification was conducted using an All‐in‐One miRNA qRT‐PCR Detection Kit (GeneCopoeia, Rockville, MD, USA). GAPDH and U6 were used for normalization. The relative expression of RNAs was calculated using the relative quantification 2^−ΔΔCt^ method. Primer sequences are listed in Table 1.

**Table 1:** Primer sequences of qRT‐PCR

Gene name	Primer Sequences(5′‐3′)
TCONS‐00106987	Forward: GCCAAGACACAGGTAATGCACAAC Reverse: AGCAACTTGCATCTGGAGATCGAC
KCNJ2	Forward: GCAGGAGCCGCTTCGTGAAG Reverse: CCAGGCAGAAGATAACCAGCATCC
GAPDH	Forward: ACTTCGGCATTGTGGAGG Reverse: GGAGGCAGGGATGATGTTCT
miR‐26a‐5p	CGCGTTCAAGTAATCCAGGATAGGCT
miR‐26b‐5p	GCGCGTTCAAGTAATTCAGGATAGGT
U6	CTCGCTTCGGCAGCACA

### 2.8 Rapid‐amplification of cDNA ends

Total RNA was extracted from rabbit atrial tissues using TRIzol reagent (Invitrogen, Carlsbad, CA, USA). First‐strand cDNA synthesis was performed using the SMARTer RACE 5′/3′ Kit and 3′ ‐Full RACE Core Set with PrimeScript Rtase (TAKARA, Dalian, China). cDNA amplification was then performed using Tks Gflex DNA Polymerase (Takara, Dalian, China). Amplified PCR products were evaluated by 1% agarose gel electrophoresis, and the DNA was sequenced after purification from gels using a MiniBEST Agarose Gel DNA Extraction Kit (TAKARA, Dalian, China). The sequences of primers used in the experiments are listed in Table 2.

**Table 2:** Primer sequences of RACE

	Primer Sequences(5′‐3′)
3’RACE	F1: AATAGTTTTCTATCCCTCTAAGAG F2: CATTTTAAGGATGTCACTATAGCA
5’RACE	R1: GGAGATCGACCCCAGATATTCACC R2: AAACAGAAAAGAAAAATTGCCATT
PCR	Forward: AGAAAGAGGGAGGGAAATCTTTCAAC Reverse: ATATAAAAATATGTACAGTCCATG

### 2.9 RNA fluorescence in situ hybridization

CY3‐labelled probes specific for TCONS‐00106987 were used for RNA‐FISH experiments involving primary cardiomyocytes fixed on slides with 4% paraformaldehyde. Hybridization was then performed by incubating the cells with the probes at 20°C for 12 hours. Images were obtained using a fluorescence microscope (Leica Microsystems DMi8, Germany). The slides were counterstained using 4′,6‐diamidino‐2‐phenylindole (DAPI).

### 2.10 Lentiviruses construction

Short hairpin RNA (shRNA) lentiviruses were designed by subcloning an oligo siRNA targeting TCONS‐00106987 into a lentiviral expression vector GV 493 (hU6‐MCS‐CBh‐gcGFP‐IRES‐puromycin) (GeneChem, Shanghai, China). A negative control shRNA was used to control for off‐target and nonspecific effects of shRNA treatment. The lentiviral overexpression vectors were developed by subcloning TCONS‐00106987/miR‐26a/miR‐26b into GV502(RV‐SV40‐EGFP‐IRES‐puromycin) (GeneChem, Shanghai, China). Following digestion and ligation, clones were selected and verified for the inserted sequence. Then we co‐transfected the shuttle vectors and packaging plasmids, pHelper 1.0 (containing *Gag*, *Pol*, *Rev*) and pHelper 2.0 (containing *VSV‐G*), into 293T cells. The lentiviruses were collected 48 hours after transfection. The titre of lentiviruses was more than 1 × 10^8^ TU/mL after concentration and purification. The shRNA sequences are listed in Table 3.

**Table 3:** Sequences of lncRNA inhibition lentiviruses and miRNA mimics

Name	Sequences
inhibitor1	Sense: GCAAAUAUUUACCAGCCUUTT Antisense: AAGGCUGGUAAAUAUUUGCTT
inhibitor2	Sense: GGCAUAGAUUCCUGGUUAUTT Antisense: AUAACCAGGAAUCUAUGCCTT
inhibitor3	Sense: GCACAAACCAUGGACUGUATT Antisense: UACAGUCCAUGGUUUGUGCTT
miRNA‐26a‐5p mimics	Sense: UUCAAGUAAUCCAGGAUAGGCU Antisense: CCUAUCCUGGAUUACUUGAAUU
miRNA‐26b‐5p mimics	Sense: UUCAAGUAAUUCAGGAUAGGU Antisense: CUAUCCUGAAUUACUUGAAUU
negative control	Sense: UUCUCCGAACGUGUCACGU Antisense: ACGUGACACGUUCGGAGAA

### 2.11 Cell transfection

Primary cardiomyocytes were cultured at a density of 5** × **10^4^ cells/well in six‐well plates and randomly divided into four groups. The NC group was cultured with a negative control lentivirus. The LV‐TCONS‐00106987 group was cultured with a lentivirus overexpressing TCONS‐00106987, the LV‐TCONS‐00106987 inhibition group was cultured with three lentiviruses targeting TCONS‐00106987, and the co‐infection group was cultured with a lentivirus overexpressing TCONS‐00106987 and lentivirus overexpressing miR‐26. After the cells were grown to 20%‐30% confluence, the corresponding viruses were transfected into each group in the presence of HitransG P (GeneChem, Shanghai, China).

### 2.12 In vivo infection

Sixteen adult New Zealand white rabbits of either sex were randomly allocated into four groups (n = 4 per group) according to the cell transfection groups. After anaesthetizing the rabbits using an injection of intravenous pentobarbital Na (30 mg/kg), the right atria were exposed and fixed. Then, 1 × 10^7^ virus particles of each lentivirus were injected into 10 different atrial sites. At 14 days after infection, the right atria were removed and frozen in liquid nitrogen for use in RNA and protein quantification analyses. AERP and AF inducibility were measured prior to infection and at 14 days after infection. The procedures are described in the ‘In vivo electrophysiological study’.

### 2.13 Whole‐cell patch‐clamp recordings

Cardiomyocytes were used to determine I_K1_ after lentivirus infection. Whole‐cell patch‐clamp recordings were performed using an amplifier (EPC10, HEKA, Germany). Under an inverted microscope (TE300, Nikon), a glass electrode micromanipulator (MP225, Sutter Instruments) was used to contact the recording electrode to the cell, and then a negative pressure was applied to promote the formation of the GΩ seal of the cell. After the GΩ seal was formed, rapid capacitance compensation was performed, and negative pressure was then continuously applied to suction the cell membrane and form the whole‐cell recording mode. For recordings of IK1, the pipette solution contained 130 mmol/L K‐aspartic, 5 mmol/L MgCl_2_, 10 mmol/L Na‐ATP, 5 mmol/L EGTA and 10 mmol/L HEPES (pH 7.2 with KOH). The external Tyrode's solution contained 137 mmol/L NaCl, 4 mmol/L KCl, 1.8 mmol/L CaCl_2_, 1 mmol/L MgCl_2_, 10 mmol/L HEPES and 10 mmol/L glucose (pH 7.4 with NaOH). The experiments were conducted at room temperature. IK1 was recorded with 400‐ms square‐wave pulses to voltages ranging from −120 mV to +20 mV with a holding potential of −50 mV at a frequency of 20 kHz. To control for diversities in cell size, currents were standardized to the membrane capacity and expressed as current density (pA/pF).

### 2.14 Western blot analysis

The protein levels of KCNJ2 in rabbit atria and cardiomyocytes were measured by Western blotting. The protein content of the lysates was determined using a BCA kit (Beyotime, Shanghai, China). Protein lysates were separated using 10% SDS‐PAGE and then transferred to a polyvinylidene fluoride membrane (Millipore, MA). After blocking with 5% skim milk powder for 1 hour, the membranes were incubated with primary antibodies specific for KCNJ2 (1:500, Proteintech, China) or GAPDH (1:1000, Proteintech, China) at 4°C overnight. The membranes were then washed and incubated with secondary antibody for 2 hours at room temperature. Quantification was performed using ECL chromogenic substrate (Millipore, MA) on a densitometer.

### 2.15 Dual‐luciferase reporter assay

The putative miR‐26 binding sites in the 3′ UTR of KCNJ2 and TCONS‐00106987 (both wild‐type and mutant forms) were cloned into pmir‐GLO luciferase vectors. The luciferase vectors were co‐transfected with miR‐NC or miR‐26 mimics into HEK293t cells using Lipofectamine 2000 (Invitrogen, Carlsbad, USA). Forty‐eight hours after transfection, cells were lysed and assayed for luciferase and Renilla production using the Dual‐Luciferase Assay Kit (Promega, USA) on a GloMax^™^ 20/20 Luminometer. The relative luciferase signal values were normalized according to the matched Renilla values.

### 2.16 Statistical analysis

SPSS Statistics 20 software (IBM Corp, Armonk, NY) was used for statistical analyses. Data are presented as the mean ± standard error of the mean (SEM). Two‐tailed unpaired Student's t tests were used to detect the significance of mean values between two groups, while one‐way analysis of variance (ANOVA) was performed for comparison among multiple groups. A *P* < .05 was considered statistically significant, and *P* < .01 was considered highly statistically significant.

## 3 RESULTS

### 3.1 The expression of TCONS‐00106987 is significantly up‐regulated during AF

**Figure 1:**
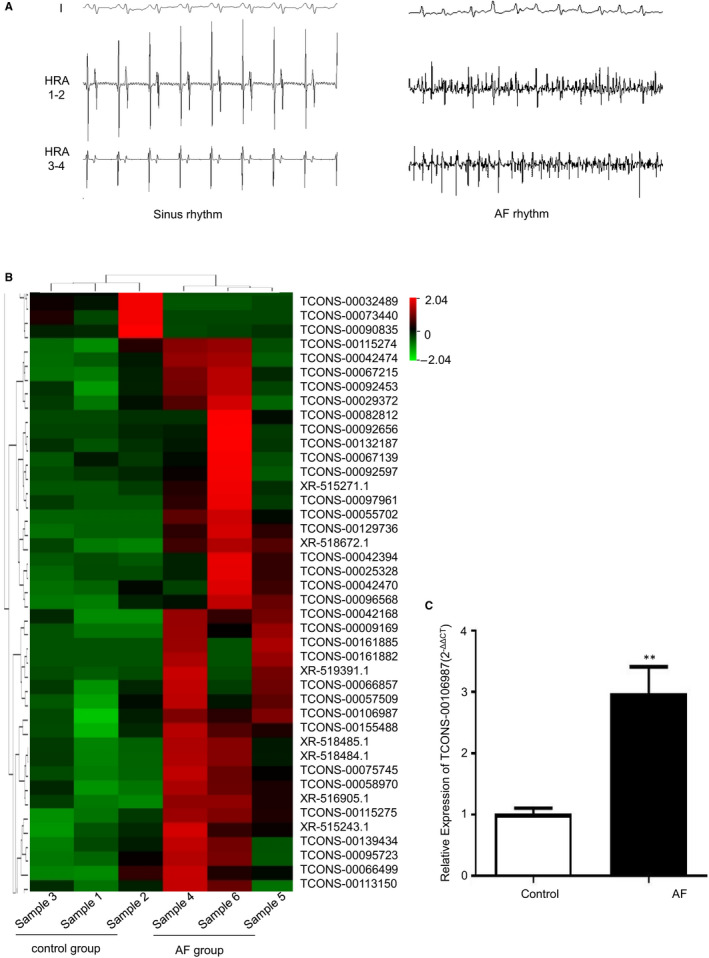
Analysis of differentially expressed lncRNAs in the AF model. A, Electrocardiograph of sinus rhythm and AF. B, Heatmap of differentially expressed lncRNAs between the control and AF groups. C, qRT‐PCR analysis of rabbit atria TCONS‐00106987 expression (n = 5). *P < .05 and **P < .01

**Figure 2:**
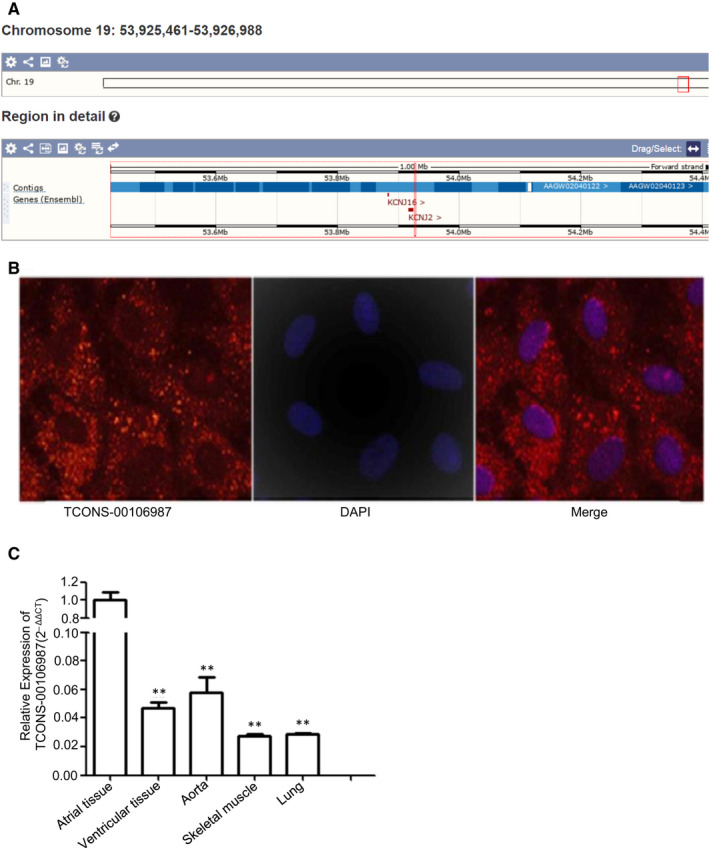
Identification of TCONS‐00106987. A, Sequence analysis of TCONS‐00106987 using Ensemble BLAST. B, The localization of TCONS_00106987 in cardiomyocytes as assessed by RNA‐FISH. TCONS_00106987 is in red and DAPI is in blue. Magnification 400×. C, Relative expression levels of TCONS_000106987 in different rabbit tissues according to qRT‐PCR. *P < .05 and **P < .01

AF models were successfully established after 7 days of pacing (Figure 1A). AERP significantly decreased and AF inducibility clearly increased in the AF group compared to that in the control group.^18^ High‐throughput sequencing was performed to detect lncRNAs and mRNAs. A total of 1364 DETs were identified as differentially expressed lncRNAs, and of these, 1110 were down‐regulated and 254 were up‐regulated. A heatmap describing the changes in lncRNAs is shown in Figure 1B. To find lncRNAs that might be related to atrial electrical remodelling, we confirmed the location of all differentially expressed lncRNAs and predicted their adjacent/localized genes through Ensemble BLAST. The results showed that the location of TCONS‐00106987 is close to *KCNJ2*. The main background inward‐rectifier current I_K1_, which determines resting potential and terminal phase‐3 repolarization in myocardium, is regulated by KCNJ2.^22^ Previous studies revealed that up‐regulation of *KCNJ2* expression exerts a great influence on atrial electrophysiology during AF.^23^ So, lncRNA TCONS‐00106987 was selected for further investigation. We found that the expression of TCONS‐00106987 was markedly increased in the AF group, and we confirmed this result using qRT‐PCR (Figure 1C). To confirm the authenticity of the lncRNA, we performed 5′ RACE and 3′ RACE to explore the full length of TCONS‐00106987, and we determined through Ensemble BLAST that the transcript is located on the forward strand of chromosome 19:53925461‐53926988 (OryCun 2.0) and possessed no protein‐coding loci (Figure 2A). RNA‐FISH results revealed that TCONS‐00106987 was primarily spread throughout the cytoplasm of cardiomyocytes (Figure 2B). Finally, the qRT‐PCR results indicated that TCONS‐00106987 was highly expressed in atria compared to its expression in other tissues (Figure 2C).

### 3.2 Modulation of TCONS‐00106987 expression affects cardiac electrophysiology

**Figure 3:**
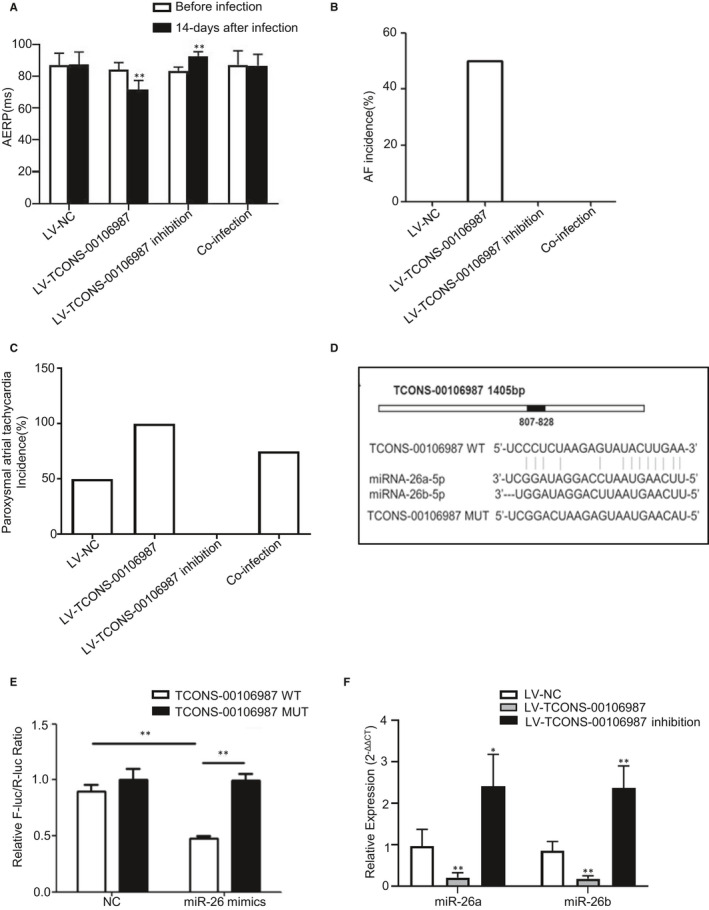
Modulation of TCONS‐00106987 and miR‐26 expression affects cardiac electrophysiology and the interaction between TCONS‐00106987/miR‐26. A, Changes in AERP before and 14 d after lentivirus infection. B, C, The AF and paroxysmal atrial tachycardia incidence measured after 14 d of infection. *P < .05 and **P < .01. D, The potential binding sites between TCONS‐00106987 and miR‐26, and the constructed mutant binding sites. E, Results of the dual‐luciferase reporter gene assay. F, Relative expression of miRNA‐26a and miR‐26b measured after infection with TCONS‐00106987 overexpression and inhibition viruses in cardiomyocytes. *P < .05 and **P < .01

At 14 days post‐infection of rabbit right atria with LV‐NC, LV‐TCONS‐00106987 and LV‐TCONS‐00106987 inhibition viruses, AERP was markedly decreased in the LV‐TCONS‐00106987 group (71.7 ± 2.8 vs 84.2 ± 2.2, *P < .01*) and was significantly increased in the LV‐TCONS‐00106987 inhibition group (92.5 ± 1.4 vs 83.3 ± 1.2, *P < .01*) compared to that of the LV‐NC group (87.5 ± 3.9 vs 86.8 ± 3.8) (Figure 3A). The AF inducibility was also altered. In the NC group, only paroxysmal atrial tachycardia was triggered in two rabbits and in the LV‐TCONS‐00106987 group, AF was induced in two out of four animals, while paroxysmal atrial tachycardia was observed in all four animals. In the LV‐TCONS‐00106987‐inhibition group, no paroxysmal atrial tachycardia was detected and no AF was induced (Figure 3B and C).

### 3.3 TCONS‐00106987 directly interacts with miR‐26

LncRNAs that are abundant in the cytoplasm typically function through ceRNA mechanisms; we examined if TCONS‐00106987 regulates AF development via this mechanism. We implemented a research for miRNAs that have complementary base pairing with *KCNJ2* using TargetScan (http://www.targetscan.org/vert_72/). MiR‐26 was screened out since it has the same pairing targets with both TCONS‐00106987 and *KCNJ2*. To determine the direct interaction between TCONS‐00106987 and miR‐26, we mutated the miR‐26 binding site in TCONS‐00106987 to generate a Luc‐TCONS‐00106987‐mut (Figure 3D). Functional (miR‐26 mimics) and negative control (mimics‐NC) miRNA analogs were also constructed. Luciferase reporter gene assay results showed that the signal generated by the TCONS‐00106987‐wt reporter was inhibited by miR‐26, while the TCONS‐00106987‐mut reporter signal was not affected (Figure 3E). This indicated that miR‐26 directly targets TCONS‐00106987 at the predicted binding site. Additionally, miR‐26 levels were decreased in response to TCONS‐00106987 overexpression, and TCONS‐00106987 silencing led to an increased level of miR‐26 in cardiomyocytes (Figure 3F).

### 3.4 TCONS‐00106987 regulates KCNJ2 expression via competitive binding with miR‐26

**Figure 4:**
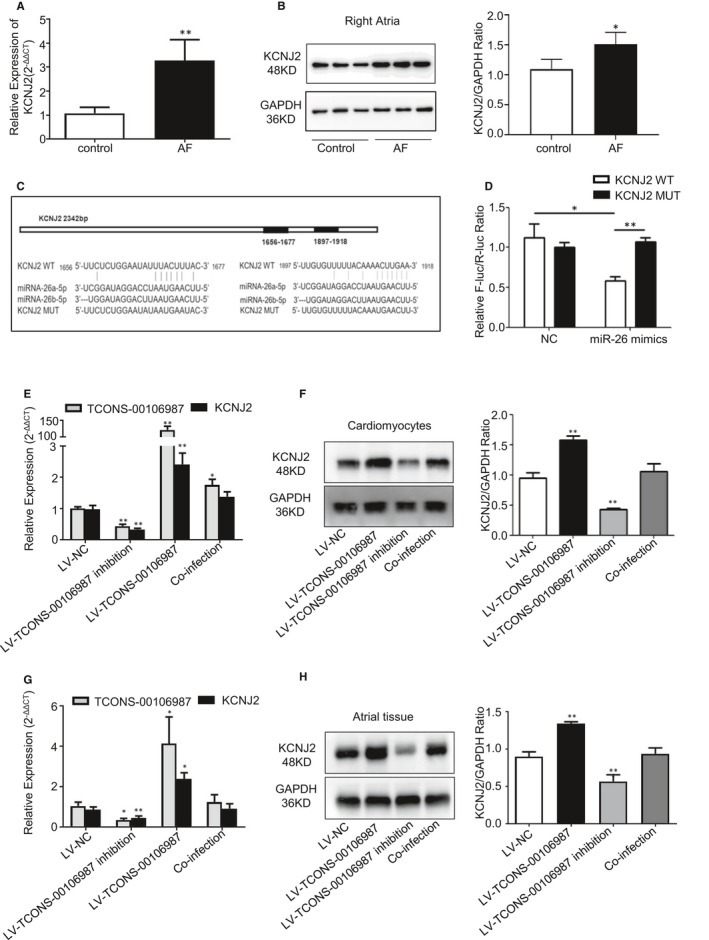
TCONS‐00106987 functions as a miR‐26 sponge to promote KCNJ2 expression in vitro and in vivo. A, Relative expression of KCNJ2 in atrial tissues between control and AF models. B, Expression of KCNJ2 in control and AF models as measured by Western blot. C, Potential binding sites between miR‐26 and KCNJ2, and the constructed mutant binding sites. D, The dual‐luciferase reporter gene assay results for miR‐26 and KCNJ2. E, F, The mRNA and protein expression of KCNJ2 after infection with viruses in cardiomyocytes. G, H, The expression levels of KCNJ2 after 14 d of infection with viruses in the right atria. *P < .05 and **P < .01

Bioinformatics analysis of the full‐length lncRNA revealed that *KCNJ2* was the neighbouring gene of TCONS‐00106987 (Figure 2A). Previous researches have shown that Kir2.1 expression and I_K1_ density were increased in patients with AF,^24^ and miR‐26 functions as an AF suppressor by targeting *KCNJ2*.^25^, ^26^ Compared to the non‐AF group, the expression of *KCNJ2* was elevated at both the mRNA and protein levels in the atria of AF models in our study (Figure 4A,B). Thus, we hypothesized that a functional relationship exists between TCONS‐00106987, miR‐26 and *KCNJ2*.

To study this possibility, the *KCNJ2* 3′ UTR wild‐type or mutant fragments were cloned into pmir‐GLO luciferase vectors (Figure 4C). The luciferase reporter gene assay results revealed that *KCNJ2* 3′ UTR is a direct target of miR‐26, and this is supported by the change in luciferase reporter activity between the wild and mutant groups (Figure 4D). Then, we co‐infected LV‐TCONS‐00106987 and LV‐miR‐26 viruses both in vitro and in vivo. The results indicated that the mRNA and protein expression levels of *KCNJ2* were significantly up‐regulated following treatment with LV‐TCONS‐00106987 viruses, and these levels were remarkably decreased following treatment with LV‐TCONS‐00106987 inhibition viruses. Furthermore, miR‐26 overexpression rescued the up‐regulation of *KCNJ2* that was induced by TCONS‐00106987 (Figure 4E,F,G,H).

### 3.5 TCONS‐00106987 promotes electrical remodelling by up‐regulating the expression of KCNJ2 as a ceRNA

**Figure 5:**
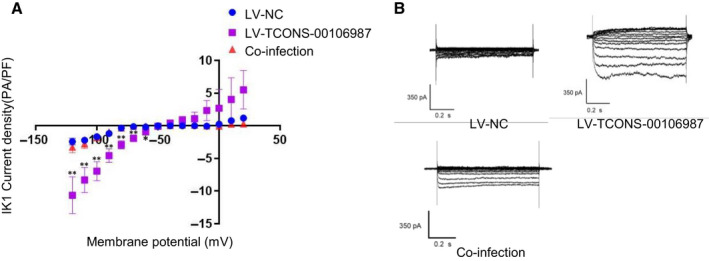
A, B, The Whole‐cell patch‐clamp recording results of cardiomyocytes after lentivirus transfection. (n = 6) *P < .05 and **P <.01

To validate the mechanisms by which TCONS‐00106987 affects atrial electrical remodelling, we co‐transfected LV‐TCONS‐00106987 virus and LV‐miR‐26 virus into cardiomyocytes and rabbit right atria. The results of whole‐cell patch‐clamp recording showed that overexpression of TCONS‐00106987 significantly elevated the density of I_K1_ current in cardiomyocytes and that co‐infection with miR‐26 can neutralize this effect (Figure 5A and B). Additionally, at 14 days after infection of rabbit atria, no significant AERP change was observed in the co‐infection group (86.7 ± 3.5 vs 87.1 ± 4.4) (Figure 3A) and this was in contrast to results observed from the LV‐TCONS‐00106987 group. Moreover, only paroxysmal atrial tachycardia was triggered in three rabbits, while no AF induction was induced (Figure 3B and C). The above results collectively suggested that TCONS‐00106987 promotes electrical remodelling in a miR‐26/*KCNJ2*‐dependent manner.

## 4 DISCUSSION

In this study, we examined the expression profiles of lncRNAs in AF and non‐AF rabbit models. We identified a novel lncRNA, TCONS‐00106987, and its role in promoting AF by triggering electrical remodelling. Mechanistically, TCONS‐00106987 displays decoy activity for miR‐26, and in doing so, it up‐regulates Kir2.1 subunits encoded by the gene *KCNJ2* in a molecular circuitry, ultimately leading to the up‐regulation of inward‐rectifier K^+^‐current.

The currently available medical treatments for AF primarily include pharmacotherapy and radiofrequency ablation, both of which possess limited efficacy.^2^ Thus, new mechanistic knowledge is essential for therapeutic innovation. It is generally accepted that the noncoding portion rather than the coding portion of the genome may explain the complexity present within higher eukaryotes.^27^ Previous studies have shown that lncRNA disorders are associated with a number of heart diseases.^13^, ^14^, ^15^, ^16^ Recently, Ruan *et al* investigated the expression profiles of lncRNAs in AF patients,^28^ and Xu Y *et al* detected the expression levels of lncRNAs during AF in elderly patients.^29^ Their studies suggested the relevance of lncRNAs in the pathogenesis of AF. However, the specific functions and regulatory mechanisms of lncRNAs in AF have not been previously described. To further study the relationship between lncRNAs and AF pathogenesis, we generated AF models through the use of atrial tachypacing. The high‐throughput RNA‐seq results indicated that the differentially expressed lncRNAs possessed a specific lncRNA expression profile in the AF models. After a series of bioinformatics analyses, the up‐regulated lncRNA TCONS‐00106987 was selected for in‐depth analysis. To confirm the potential role of TCONS‐00106987 in AF, we conducted gain‐ and loss‐of‐function experiments. After 14 days of lentiviral infection, AERP was significantly shortened and AF inducibility was increased in the TCONS‐00106987 overexpression group, while there was no significant difference in the negative control group. TCONS‐00106987 repression resulted in the opposite effects. These results indicated a potential role of TCONS‐00106987 in the regulation of atrial electrophysiology during AF. As electrophysiological changes often occur in the early term of AF,^6^ increased expression of lncRNAs may act as a possible indicator of AF.

Recent data suggest that coding and noncoding RNAs can regulate each other by competitive binding to miRNAs, and these molecules have been referred to as competing endogenous RNA (ceRNA). ceRNAs can protect target RNAs from repression by secluding miRNAs.^30^ According to bioinformatics analyses, we found that TCONS‐00106987 contains putative miR‐26 binding sites. Previous studies have demonstrated that miR‐26 is down‐regulated in the atrial tissues of AF animals and patients.^26^ Our luciferase reporter assay results showed that the expression of TCONS‐00106987 was significantly inhibited when functional miR‐26 mimics were introduced into cells, and our findings indicated that mutating the relevant sequence in the TCONS‐00106987/miR‐26 base pairing regions could rescue this inhibition. Moreover, the expression of miR‐26 was decreased after overexpression of TCONS‐00106987, and silencing of TCONS‐00106987 resulted in increased expression of miR‐26 in cardiomyocytes, indicating that TCONS‐001069897 expression was negatively correlated with miR‐26.

Atrial electrophysiological peculiarities are controlled by pumps, ion channels, and exchangers, and any of these can be modulated by atrial remodelling.^4^ Electrical remodelling is significantly characterized by increased inward‐rectifier K^+^ current, decreased L‐type Ca^2+^‐current and abnormal distribution of the gap junction connexin hemichannels.^6^ *KCNJ2* encodes the alpha subunits of Kir2.1. The main background cardiac inward‐rectifier K^+^ current I_K1_, which determines the resting potential and terminal phase‐3 repolarization, is composed primarily of Kir2.1. Up‐regulation of I_K1_ exerts a great influence on atrial electrophysiology, and I_K1_ is increased in patients with AF.^24^, ^25^, ^31^, ^32^, ^33^, ^34^ Previous studies revealed that the down‐regulation of miR‐26 may promote AF by targeting *KCNJ2*.^26^ Our previous studies have shown that lncRNAs can modulate the electrical remodelling of AF by targeting the calcium channel‐encoding gene *CACNA1C*.^18^ Based on this, we considered that TCONS‐00106987 could affect *KCNJ2* expression by endogenously competing with miR‐26. The luciferase reporter assay results suggested that *KCNJ2* is a target gene of miR‐26. Further co‐infection experiments in vitro and in vivo revealed that miR‐26 could partially reverse the up‐regulation of *KCNJ2* caused by TCONS‐00106987 overexpression. Additionally, miR‐26 mitigated the electrophysiological changes that resulted from increased expression of TCONS‐00106987. These results verified the possibility that TCONS‐00106987 promotes the initiation and progression of AF by acting as a ceRNA to regulate the expression of *KCNJ2*.

Taken together, our results demonstrated the role and mechanisms of the novel lncRNA TCONS‐00106987 in AF. As lncRNAs function relatively upstream in the intracellular signalling network, they regulate gene expression primarily at the transcriptional and epigenetic levels rather than at a translational level.^35^ Furthermore, the use of RNA as a therapeutic medium would rapidly modulate regulatory functions without protein translation, as RNA structures may be deployed faster and easier than can proteins.^36^, ^37^ Additionally, due to the enhanced tissue specificity of lncRNAs and the ability of lncRNAs to function as ceRNAs to regulate miRNA/mRNA, therapeutic targeting of lncRNAs may result in fewer off‐target effects than other therapeutic strategies.^36^ These characteristics highlight the idea that therapeutic approaches that target lncRNAs may be faster and more efficient than methods that target proteins. Accordingly, we suggest that lncRNAs could be utilized as effective therapeutic targets for AF in future.

## 5 LIMITATIONS

The homology of lncRNAs between rabbits and humans is unknown, and the mechanisms by which AF regulates lncRNA expression should be explored further.

## CONFLICT OF INTEREST

The authors confirm that there are no conflicts of interest.

## AUTHOR CONTRIBUTION

**Juanjuan Du:** Data curation (lead); Investigation (lead); Methodology (equal); Software (equal); Writing‐original draft (lead). **Zhan Li:** Formal analysis (equal); Methodology (equal); Supervision (equal); Writing‐review & editing (equal). **Xiao Wang:** Writing‐review & editing (lead). **Jianhua Li:** Data curation (equal); Formal analysis (equal); Investigation (supporting); Software (equal). **Donglu Liu:** Investigation (supporting); Software (equal); Validation (equal). **Ximin Wang:** Methodology (supporting); Supervision (equal); Visualization (equal); Writing‐review & editing (supporting). **Jinqiu Wei:** Resources (equal); Validation (equal). **Shenzhou Ma:** Software (supporting); Supervision (equal). **Yujiao Zhang:** Funding acquisition (equal); Supervision (supporting); Writing‐review & editing (equal). **Yinglong Hou:** Conceptualization (lead); Funding acquisition (equal); Writing‐review & editing (supporting).

## DATA STATEMENT

The data that supports the findings of this study are available in the [Link], [Link], [Link] of this article.

## Supporting information

File S1

File S2

File S3
