# Supplementary material for: Long noncoding RNA TCONS‐00106987 promotes atrial electrical remodelling during atrial fibrillation by sponging miR‐26 to regulate KCNJ2
Source: J Cell Mol Med. 2020 Sep 20;24(21):12777–88. doi: 10.1111/jcmm.15869 (PMC7687017; doi:10.1111/jcmm.15869)
Supplement: Supplementary file 2 — File S2 [file JCMM-24-12777-s002.pdf]

Supplementary Figure 1

A

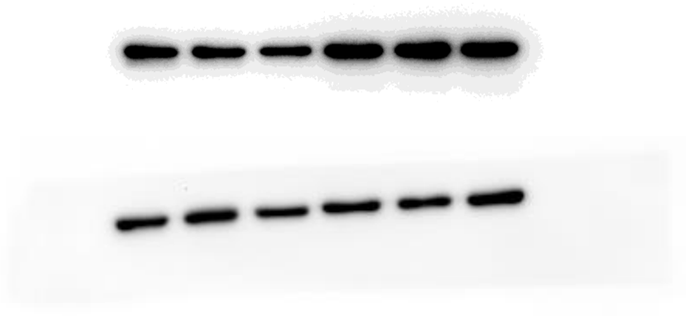

C

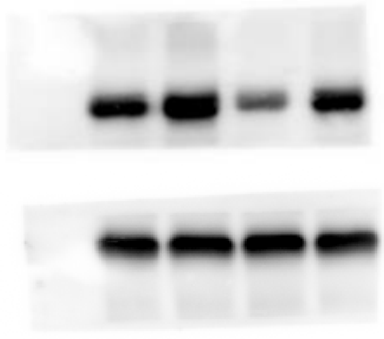

B

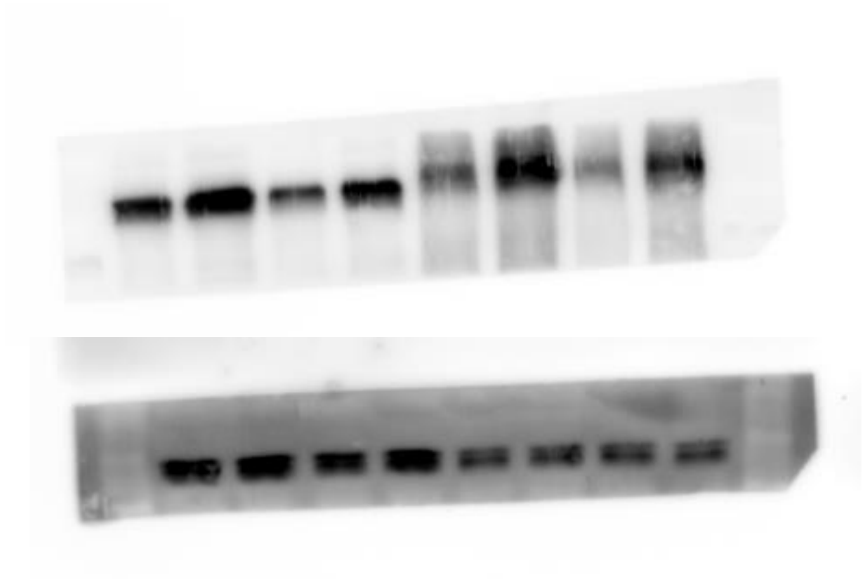

Supplementary Figure 1. (A)(B)(C) : the original western blot images of Figure(4B)(4F)(4H)
