# Supplementary material for: Long noncoding RNA TCONS‐00106987 promotes atrial electrical remodelling during atrial fibrillation by sponging miR‐26 to regulate KCNJ2
Source: J Cell Mol Med. 2020 Sep 20;24(21):12777–88. doi: 10.1111/jcmm.15869 (PMC7687017; doi:10.1111/jcmm.15869)
Supplement: Supplementary file 3 — File S3 [file JCMM-24-12777-s003.docx]

Full length sequence of TCONS-00106987

| 5’-3’：GGTATGGGGGCAAGAAAGAGGGAGGGAAATCTTTCAACTTATTTCTGAAAAGAGAAAAAAATTAAAATTTCTGGTGCACAGGTTTGTTTTTTTTTTTTTCAAGAAAATTTTGCAGAAGCTATGTTTTTAAAGTGTACATTTTATAAAGTTTATCAGATATTTTCATATTTAAAGCCAAATGTAAATAGAAGTCTGTAAAGGAAAAAATTGCCATAGAAAGTATGATTTCAGTGCAGCAATTTCTGAGAGCTAGCACCTATATGCTACCGGTTAGCATGGTTTTAGCAAATATTTACCAGCCTTATAAGGTTCGTATTGCTATGTTCTTCTGTTATTTATTTCAGCATGGACTGTTCATTTGAAAGCTTTTTCTAGTTATTGGCATTTTAATAGTTATAAGCTTTAAATGGCAATTTTTCTTTTCTGTTTTTCCTTTTATTTTTTTTTTTTTCATCAAGAGCCAAGACACAGGTAATGCACAACATTTATTGCTGCGTTTTACCTTCAAAACATTTGTCCTTATTGACTGGGTCTCCTTTACTGGTGCACACATGTCACTAGAATGCAGATGGAAGGGACTCACGGTGAATATCTGGGGTCGATCTCCAGATGCAAGTTGCTTCTGTATTGTAAGCAAACCCCTAATAACTTACCTAGGATGTAATCCCTTTTAAAGAATGTTTGCCCATATCTGGATGGGCACTATATTTTTGGGGGAAGGCATAGATTCCTGGTTATCCTATTTTTAAATAAAAGGTAGACAAAGTGAATTCTATTTTGATTATTGAGAAAGGAATAGTTTTCTATCCCTCTAAGAGTATACTTGAATCAGACATTTTAAGGATGTCACTATAGCACTGTAGTTGTTTCCAAATTCCTAAGAAAGGTTTTTTACGTTGTTTTTGTTCTATTAATGCATTATTTCTTCCCTACCCTACTATTCTTCCCTATCCACTTCCATCCCAAATCCTGATGTTTATGTGATGAGCTGTCCCCCAAATCATGTTCGCCTTGCAAGTAGTAAGTGTCACTTCATATTTCATAGTAAACCACTCAGTAAACACAAAAAGCTTGTCCTGAGAAGTTGGGTGAGTTCTTTTTCACTCTGTGTCCAACAATGTTAAGGTGGGAAAAAAAAAAGTGTTGCATAGTCACCTCTCACCTCAACCTTTGGCAAACTAGAATTTCTTTTCTAGTAATGTTAGGTTTCTGCTCCAGGTTCTAGAGAGTGGAGCAATCCTGGTCTTTGACAATCATGATAGTTATTATTTTCCCATTGACCATCTTTTTGTATCTAAAGTCTTCCTATTGTACTGCACAAACCATGGACTGTACATATTTTTATATGTTATGTCTTATTTTATTATTTCTAAATAAAAAATTGAAAACAGGCAAAAAAAAAAAA |
| --- |
